# The signal intensity variation of multiple sclerosis (MS) lesions on magnetic resonance imaging (MRI) as a potential biomarker for patients’ disability: A feasibility study

**DOI:** 10.3389/fnins.2023.1145251

**Published:** 2023-03-13

**Authors:** Sam Sedaghat, Hyungseok Jang, Jiyo S. Athertya, Martin Groezinger, Jody Corey-Bloom, Jiang Du

**Affiliations:** ^1^Department of Radiology, University of California, San Diego, San Diego, CA, United States; ^2^University Hospital Heidelberg, Heidelberg, Germany; ^3^Department of Neurosciences, University of California, San Diego, San Diego, CA, United States; ^4^Department of Bioengineering, University of California, San Diego, San Diego, CA, United States; ^5^Radiology Service, Veterans Affairs San Diego Healthcare System, San Diego, CA, United States

**Keywords:** multiple sclerosis, EDSS score, biomarker, MRI, disability

## Abstract

**Introduction:**

Although many lesion-based MRI biomarkers in multiple sclerosis (MS) patients were investigated, none of the previous studies dealt with the signal intensity variations (SIVs) of MS lesions. In this study, the SIVs of MS lesions on direct myelin imaging and standard clinical sequences as possible MRI biomarkers for disability in MS patients were assessed.

**Methods:**

Twenty seven MS patients were included in this prospective study. IR-UTE, FLAIR, and MPRAGE sequences were employed on a 3T scanner. Regions of interest (ROIs) were manually drawn within the MS lesions, and the cerebrospinal fluid (CSF) and signal intensity ratios (SIR) were calculated from the derived values. Variations coefficients were determined from the standard deviations (Coeff 1) and the absolute differences (Coeff 2) of the SIRs. Disability grade was assessed by the expanded disability status scale (EDSS). Cortical/gray matter, subcortical, infratentorial, and spinal lesions were excluded.

**Results:**

The mean diameter of the lesions was 7.8 ± 1.97 mm, while the mean EDSS score was 4.5 ± 1.73. We found moderate correlations between the EDSS and Coeff 1 and 2 on IR-UTE and MPRAGE images. Accordingly, Pearson’s correlations on IR-UTE were *R* = 0.51 (*p* = 0.007) and *R* = 0.49 (*p* = 0.01) for Coeff 1 and 2, respectively. For MPRAGE, Pearson’s correlations were *R* = 0.5 (*p* = 0.008) and *R* = 0.48 (*p* = 0.012) for Coeff 1 and 2, respectively. For FLAIR, only poor correlations could be found.

**Conclusion:**

The SIVs of MS lesions on IR-UTE and MPRAGE images, assessed by Coeff 1 and 2, could be used as novel potential MRI biomarkers for patients’ disability.

## 1. Introduction

Multiple sclerosis (MS) is a chronic autoimmune disease of the central nervous system (CNS) that is characterized by demyelination and axonal loss (Oh et al., 2018). The disease mainly affects young adults between 20 and 40, and women are at an approximately three times higher risk (Amato et al., 2018; Oh et al., 2018). Although diagnostics and therapy have improved in recent decades, MS remains one of the leading causes of neurological disability in young patients (Compston and Cole, 2008).

The most popular grading system for evaluating the severity of disability in MS patients is the expanded disability status scale (EDSS) (Kurtzke, 1983; Meyer-Moock et al., 2014). The EDSS is used to describe MS progression and illustrates an ordinal rating system ranging from 0 (normal neurological status) to 10 (death due to MS) (Kurtzke, 1983; Meyer-Moock et al., 2014).

Magnetic resonance imaging (MRI) has gained significant importance in diagnosing MS and predicting disease progression (Kaunzner and Gauthier, 2017). With rapidly increasing developments in the field of MRI, it is favorable to establish sequences that allow direct visualization of myelin. As myelin has very short T2* values of less than 1 msec (Horch et al., 2011; Wilhelm et al., 2012; Du et al., 2014a; Boucneau et al., 2018; Ma et al., 2020a,b; Jang et al., 2021; Mueller et al., 2022), the changes seen on clinical T1- or T2-weighted images are not specific to myelin, as they only depict longer T2 components. With the introduction of ultrashort echo time (UTE) MRI sequences, which have 100–1000 times shorter echo times than conventional sequences, myelin can potentially be visualized directly (Horch et al., 2011; Wilhelm et al., 2012; Ma et al., 2020b). Three-dimensional adiabatic inversion recovery UTE (IR-UTE) sequences allow for robust inversion and nulling of long T2 water components; thereby, volumetric imaging of short T2 species such as myelin with greater excitation efficiency and reduced eddy current artifacts (Du et al., 2014a; Ma et al., 2020a).

In MS disease pathology, demyelination and remyelination mainly occur in parallel within the same lesion (Filippi et al., 2019). Many studies investigated correlations between pathological changes on MRI and disability in patients with Jang et al. (2021) found a moderate correlation between the IR-UTE signal in the normal-appearing white matter (NAWM) and the EDSS (Jang et al., 2021). Other authors showed that correlations could also be found on standard clinical MRI sequences. Accordingly, an increased T2-hyperintense lesion load and a higher lesion volume may be associated with increased disability (Rudick et al., 2006).

Although many correlating studies using MRI exist, none of the previous studies investigated the signal intensity variations (SIVs) of MS lesions as potential biomarkers on direct myelin imaging and standard clinical MRI sequences.

Therefore, this feasibility study evaluated whether the SIVs of MS lesions on IR-UTE as a direct myelin imaging sequence and standard clinical sequences (FLAIR and MPRAGE) could be used as potential biomarkers for disability in MS patients assessed by the EDSS.

## 2. Materials and methods

### 2.1. Subjects

A total of 36 patients with the diagnosis of MS were included in this prospective clinical study, which was reviewed and approved by the University of California San Diego Institutional Review Board (IRB). Written informed consent was obtained from each subject in accordance with the IRB guidelines before the MRI scan. In this study, periventricular and juxtacortical MS lesions were evaluated. Due to the much lower myelin content in gray matter, which is more challenging to detect with IR-UTE sequences, we excluded cortical/gray matter, subcortical and infratentorial lesions. Also, spinal cord lesions were excluded, as our patients did not undergo cervical spine examinations. Five patients were excluded due to motion artifacts, which affected the image quality significantly. Four patients had less than two measurable lesions and were also excluded. The threshold was set at 3 mm on the short axis (see section “2.3. Imaging analysis”). A neurologist collected the EDSS score of each patient before the examination in accordance with the proposed 10-digit score with half-score steps (Kurtzke, 1983; Meyer-Moock et al., 2014).

### 2.2. Data acquisition

**FIGURE 1:**
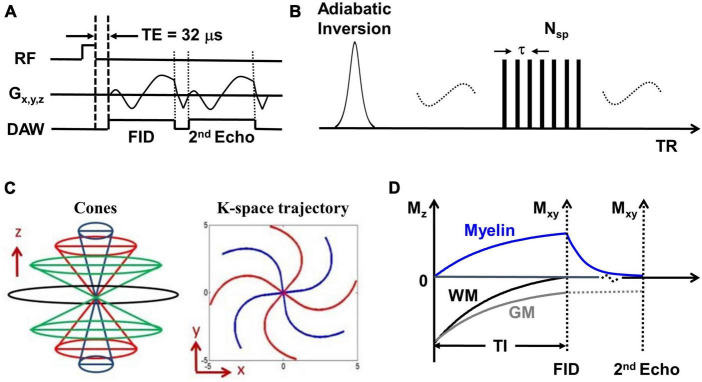
**(A)** The basic 3D UTE sequence employs a short rectangular pulse for signal excitation followed by dual-echo spiral cones sampling. **(B)** The data acquisition scheme where each adiabatic inversion recovery preparation is followed by multi-spoke (Nsp) UTE data acquisitions. **(C)** The cones data sampling strategy and k-space trajectory. **(D)** The contrast mechanism in 3D IR-UTE myelin imaging where the long adiabatic inversion pulse inverts and nulls the longitudinal magnetization of long T2 white matter. The myelin magnetization is largely saturated by the adiabatic inversion pulse due to its extremely short T2 and fast relaxation during the long adiabatic inversion process. The saturated myelin magnetization recovers fast during the inversion time (TI) and is selectively imaged by subsequent UTE free induction decay (FID) sampling. Gray matter recovers slower than white matter due to its longer T1. The residual gray matter signal is suppressed by subtracting the 2nd echo image from the first one.

The whole brain was scanned using the IR-UTE, FLAIR, and MPRAGE sequences on a 3T MR750 scanner (GE Healthcare Technologies, Milwaukee, WI). A 12-channel head coil was used for signal reception. The IR-UTE sequence employs unique k-space trajectories that sample 3D data along evenly spaced twisting paths in the shape of multiple cones. The 3D UTE cones data acquisition starts as soon as possible following a short rectangular radiofrequency (RF) pulse excitation with a minimal nominal echo time (TE) of 32 μs. For imaging of short T2 components more efficiently, multiple cones spokes are acquired after each IR preparation (Figure 1A). Short T2 components (myelin) are not inverted but largely saturated by an adiabatic inversion pulse with a longer duration than the T2 values of myelin protons. The adiabatic inversion pulse inverts long T2 components (mostly water). The 3D cones data acquisition starts at an appropriately chosen inversion time (TI) so that the inverted long T2 magnetizations approach the nulling point, leading to efficient long T2 signal suppression. The short T2 myelin magnetization recovers quickly during TI due to its short T1 relaxation time and is selectively detected by 3D UTE cones data acquisition (Du et al., 2014b). A short rectangular RF pulse is used for more efficient non-selective excitation of myelin magnetizations, which cannot be excited efficiently with conventional RF pulses due to fast relaxation (Figure 1B). A second echo detects residual long T2 signals. Efficient sampling of 3D k-space is performed from the center of k-space using spiral trajectories with conical view ordering (Figure 1C). The combination of 3D conical trajectories and multi-spoke acquisition allows time-efficient volumetric myelin imaging (Ma et al., 2018, 2020a). The TI is chosen such that the long T2 signal in white matter is nulled (Figure 1D). Consecutively, the remaining UTE signal in the white matter originates from myelin. Finally, a dual-echo subtraction is performed that reduces residual long T2 signals.

The following imaging protocol was used: (1) 3D IR-UTE: TR/TI = 1000/320 ms, dual TEs = 0.032/2.2 ms, number of spoke (Nsp) = 21, time for each spoke (tau) = 7.1 ms, flip angle (FA) = 20°, sampling bandwidth = 250 kHz, field of view (FOV) = 22 × 22 × 15.1 cm^3^, acquisition matrix = 192 × 192 × 42, scan time = 8.3 min, resolution: 1.1 × 1.1 × 3.6 mm^3^; (2) 3D T2-weighted FLAIR sequence: TR/TI = 7600/2162 ms, TE = 117 ms, FOV = 25.6 × 25.6 × 25.6 cm^3^, acquisition matrix = 256 × 256 × 256, acceleration factor = 4, scan time = 6.9 min, resolution: 1 × 1 × 1 mm^3^; (3) 3D T1-weighed MP-RAGE sequence: TR/TI = 7/450 ms, TE = 3 ms, FOV = 22 × 22 × 16 cm^3^, acquisition matrix = 256 × 256 × 136, scan time = 4.2 min, resolution: 0.9 × 0.9 × 1.2 mm^3^.

### 2.3. Imaging analysis

Two radiologists with 5 and 8 years of experience in MS diagnostics (readers) reviewed all available IR-UTE, FLAIR, and MPRAGE images with findings reached by consensus. The readers only determined clearly measurable lesions, as small lesions are prone to artifacts on IR-UTE. The cut-off in size was set at 3 mm in diameter on the short axis. For each included and evaluated lesion, a mean diameter was calculated from the length, width, and depth in mm. The readers manually drew regions of interest (ROI) in all measurable MS lesions ≥ 3 mm and the cerebrospinal fluid (CSF) within the lateral ventricles of the brain. From the signal intensities of each MS lesion and the CSF, a SI ratio (SIR) was calculated by dividing the SI of the MS lesion by the SI of the CSF. In the next step, the mean values with standard deviations (SD) of all derived SIRs per patient were calculated. Additionally, the lesions with the highest and lowest SIR were identified, and an absolute difference (AD) between these two values was calculated. To normalize SD and AD, both values were divided through the mean SIR of the lesions, and two normalized variation coefficients were built: coefficient 1 (Coeff 1) = SD/mean SIR and coefficient 2 (Coeff 2) = AD/mean SIR. Additionally, the median values of the SIRs were calculated. The distribution of the lesions around the median SIs was also determined. Accordingly, two groups were built: group 1 – lesions with SI values of more than 10% above the median SIs, group 2 – lesions with SI values of more than 10% below the median SIs. The derived Coeff 1 and 2 values were used as two independent SIV parameters. Coeff 1, Coeff 2, and median SIR were correlated with the EDSS. The same MS lesions were evaluated for all three MRI sequences, and the same described procedures were performed. The two readers were blinded to the EDSS of each patient.

### 2.4. Statistical analysis

Data were given as mean or median values with range (minimum to maximum) and additionally for mean diameter and mean EDSS with standard deviation (SD). Data in groups 1 and 2 were indicated as percentages (%) of the total number of included lesions per patient. Correlations between Coeff 1/2/median SI and EDSS were established using the Pearson’s correlation coefficient (“R”). The statistical significance for all tests was set at *p* < 0.05. Statistical analysis was done using the IBM-SPSS, version 26.0, software package (IBM, Armonk, NY, USA).

## 3. Results

A total of 27 patients were evaluated in this study. The mean diameter of the included MS lesions was 7.8 mm (Min.: 5 mm, Max.: 15 mm, SD: 1.97). 85% of the lesions were periventricular and 15% juxtacortical. The mean EDSS score was 4.5 (Min.: 2.5, Max.: 8, SD: 1.73).

### 3.1. IR-UTE

**FIGURE 2:**
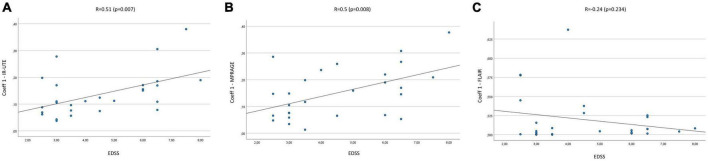
Correlation Coeff 1 in IR-UTE **(A)**, MPRAGE **(B)**, and FLAIR **(C)** images with the EDSS of MS patients.

**FIGURE 3:**
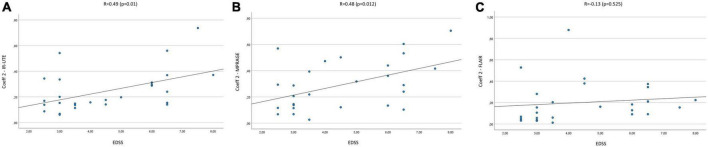
Correlation of Coeff 2 in IR-UTE **(A)**, MPRAGE **(B)**, and FLAIR **(C)** images with the EDSS of MS patients.

The mean Coeff 1 on IR-UTE was 0.14 (Min.: 0.04, Max.: 0.38), while the mean Coeff 2 was 0.25 (Min.: 0.06, Max.: 0.74). We found a moderate correlation between Coeff 1 and the EDSS with a Pearson’s correlation of *R* = 0.51 (*p* = 0.007). Pearson’s correlation between Coeff 2 and the EDSS presented with *R* = 0.49 (*p* = 0.01) (Figures 2A, 3A). The median SI of the lesions on IR-UTE was 1.17 (Min.: 0.84, Max.: 1.47). 22% of the lesions were in group 1 and 21% of the lesions were in group 2. There was no significant correlation between the median SI and the EDSS (*R* = 0.34, *p* = 0.161).

### 3.2. MPRAGE

The mean Coeff 1 on MPRAGE was 0.15 (Min.: 0.01, Max.: 0.38), while the mean Coeff 2 was 0.29 (Min.: 0.03, Max.: 0.7). The Pearson’s correlation analysis revealed a moderate correlation between Coeff 1 (*R* = 0.5; *p* = 0.008) and Coeff 2 (*R* = 0.48; *p* = 0.012) compared to the EDSS (Figures 2B, 3B). The median SI of the lesions on MPRAGE was 1.84 (Min.: 1.27, Max.: 2.58). 29% of the lesions were in group 1 and 24% in group 2. There was no significant correlation between the median SI and the EDSS (*R* = −0.31, *p* = 0.107).

### 3.3. FLAIR

The mean Coeff 1 on FLAIR was 0.52 (Min.: 0.5, Max.: 0.64), while the mean Coeff 2 was 0.2 (Min.: 0.01, Max.: 0.88). The Pearson’s correlation analysis revealed a poor correlation between Coeff 1 (*R* = −0.237, *p* = 0.234) and Coeff 2 (*R* = 0.13, *p* = 0.525) compared to the EDSS (Figures 2C, 3C). The median SI of the lesions on FLAIR was 6.96 (Min.: 2.58, Max.: 10.97). 33% of the lesions were in group 1 and 29% in group 2. There was no significant correlation between the median SI and the EDSS (*R* = 0.11, *p* = 0.576).

**FIGURE 4:**
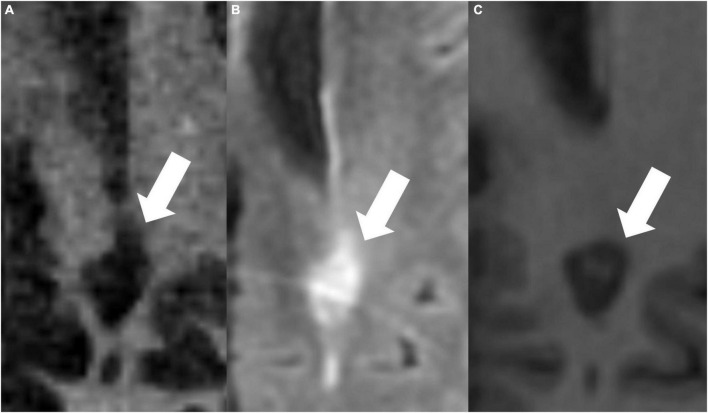
The same periventricular MS lesion (white arrows) is shown on IR-UTE **(A)**, FLAIR **(B)**, and MPRAGE **(C)** sequences, respectively.

**FIGURE 5:**
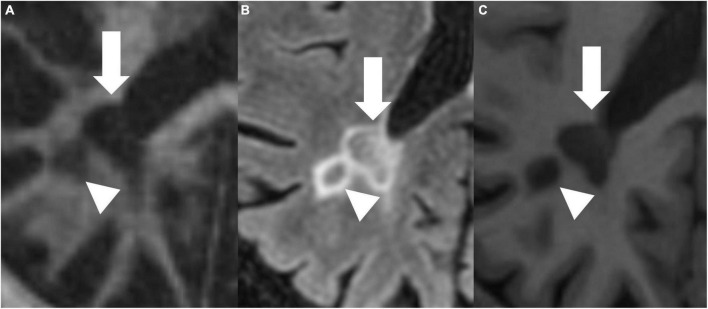
IR-UTE **(A)**, FLAIR **(B)**, and MPRAGE **(C)** images show two periventricular MS lesions (white arrow and white arrowhead) next to each other.

Figures 4, 5 present examples of MS lesions on IR-UTE, FLAIR, and MPRAGE sequences, respectively.

## 4. Discussion

In this study, we investigated the SIVs of MS lesions on IR-UTE, MPRAGE, and FLAIR images and correlated these SIVs to the EDSS. We found moderate correlations of Coeff 1 and 2 values on IR-UTE and MPRAGE images with the EDSS.

With the introduction of UTE MRI sequences, which have 100–1000 times shorter echo times than conventional sequences, myelin and its specific changes of demyelination and remyelination can potentially be visualized directly (Horch et al., 2011; Wilhelm et al., 2012; Du et al., 2014a; Boucneau et al., 2018; Jang et al., 2021; Mueller et al., 2022). The 3D IR-UTE Cones sequence allows high-contrast volumetric myelin imaging in MS patients. This sequence also has a high acquisition efficiency and reduced sensitivity to eddy currents (Ma et al., 2020a). To reduce the total scan time, 3D cones sampling trajectories are used for more efficient k-space coverage with multiple acquisition spokes per IR preparation (Ma et al., 2018, 2020a). Jang et al. (2021) studied changes in normal-appearing white matter (NAWM) on IR-UTE sequences and the correlation to the EDSS. They found a moderate correlation between the IR-UTE signal in NAWM and the EDSS (Jang et al., 2021). This is expected as brain cognitive function and behavior are highly dependent on the presence of myelin. The speed at which neuron signals are transmitted is directly related to the thickness of the myelin wrapping and neuronal myelin content (Gibson et al., 2014). Therefore, MS patients with increased disability or higher EDSS scores are expected to have lower myelin content in NAWM, as suggested by Jang et al. (2021). However, Jang et al. (2021) did not investigate the signal intensity of MS lesions (Jang et al., 2021).

Previous studies focusing on standard clinical MRI sequences also found correlations between MS lesions and the EDSS. Accordingly, increased T2-hyperintense lesions and a higher lesion volume may be associated with increased disability (Rudick et al., 2006). Especially the number of new T2-hyperintense lesions in the long term and T2-hyperintense lesion volume in the short term is associated with the EDSS (Scott et al., 2000; Fisniku et al., 2008; Minneboo et al., 2008). The so-called “black holes” (T1-hypointense lesions) display demyelination and axonal loss (Kutzelnigg and Lassmann, 2014). Also, a correlation between the number of these lesions and their volume with the EDSS was assumed (Jacobsen et al., 2014; Kaunzner and Gauthier, 2017). One non-lesion-related biomarker is cerebral atrophy, which many authors describe to illustrate one of the leading MRI markers for disability and is mainly caused by gray matter loss (Dastidar et al., 1999; Simon et al., 1999; Fisniku et al., 2008; Minneboo et al., 2008; Bonati et al., 2011). Also, multiple factor analyses were performed, stating that composite MRI measures could predict short-term disability (Bommarito et al., 2018).

Our findings suggest that the higher the EDSS of the patients, the more SIV of MS lesions is seen on IR-UTE and MPRAGE images. It is not entirely clear why the SIV correlates with the EDSS. Potential explanations should be investigated in further studies with more significant patient numbers. First, a mix of active and inactive lesions is already described to be associated with MS severity (Luchetti et al., 2018). Unfortunately, our patients did not undergo contrast-enhanced imaging. However, there is also the possibility of a “slow-burning” activity within the lesions (Zhang et al., 2014). Those lesions could show minimal inflammation without significantly enhancing the contrast agent. Also, MS lesions can present with different components and (de)myelination stages (Harrison et al., 2016). A variation or combination of various MS lesion components within one lesion or in interaction with other lesions could affect the disease severity. Further studies could therefore investigate correlations between MS lesion components and component interactions with the disease severity.

We propose two different variation coefficients for evaluating the SIVs. Both coefficients could potentially be used as novel biomarkers to estimate the EDSS in MS patients. Against this, the SIV on the FLAIR sequence is not significantly correlated to the EDSS.

Our study only included three sequences, two of which were standard clinical MRI sequences. However, many more advanced MRI imaging methods exist, such as myelin water imaging (MWI), MP2RAGE, or magnetization transfer ratio (MTR). MWI allows *in vivo* whole-brain myelin water fraction mapping and is a potent biomarker of myelin (Lee et al., 2021). A limitation of MWI is the signal-to-noise ratio requiring higher field strengths for optimal imaging (Laule et al., 2008). MP2RAGE is a magnetization-prepared rapid gradient echo sequence that is T1 weighted and allows simultaneous T1 mapping. The sequence is presumed to enable a better lesion subtype analysis and evaluate MS lesions’ activity (Kober et al., 2012). MP2RAGE is even more advantageous in deep gray matter imaging than MPRAGE (Okubo et al., 2016). Myelin content changes have often been correlated to changes in the MTR of myelin, as a loss of myelin leads to a decreased MTR. Anyways, changes in MTR are also caused by inflammation and edema, potentially masking myelin changes and posing a possible limitation to MTR myelin imaging (Vavasour et al., 2011).

As the present study is a feasibility study, there are few patient numbers. Our results should be investigated in a larger cohort within the routine clinical setting, as our patients underwent their examinations out of the routine. Further developments should also focus on automated lesion segmentation, coefficient calculation, and disability assessment to standardize our findings. As mentioned, many advanced MRI sequences are available nowadays. Therefore, the SIVs of MS lesions as potential biomarkers could be evaluated in a novel or advanced MRI sequences.

Our study has some limitations. First, we did not administer a contrast agent, which was not included in our IRB approval. Therefore, we could not determine the acuity of the MS lesions. Second, we did not take any therapy into account, as our patients underwent MRI examinations outside of the routine clinical setting. Accordingly, we could not verify the effect of treatment on our findings. Third, our patient number is relatively few. Fourth, we used a manual ROI drawing.

## 5. Conclusion

The SIVs of MS lesions on IR-UTE and MPRAGE images correlate with patients’ disability assessed by the EDSS. Therefore, SIVs on IR-UTE and MPRAGE images could potentially be novel biomarkers for patients’ disability. Both sequences and proposed variation coefficients are comparable. Further studies in larger cohorts and with an automated segmentation approach are needed to evaluate and further standardize our findings.

## Data availability statement

The datasets presented in this article are not readily available because data privacy and ethics. Requests to access the datasets should be directed to SS, samsedaghat1@gmail.com.

## Ethics statement

The studies involving human participants were reviewed and approved by the IRB of the University of California, San Diego. The patients/participants provided their written informed consent to participate in this study.

## Author contributions

SS, HJ, and JD: conceptualization, funding acquisition, resources, and visualization. SS, HJ, JC-B, and JD: data curation. SS, HJ, JA, MG, and JC-B: formal analysis. SS, HJ, MG, and JC-B: investigation. SS, HJ, MG, and JD: methodology. SS and JD: project administration. HJ and JD: software and supervision. SS, HJ, JA, and MG: validation. SS, HJ, JA, MG, and JD: writing—original draft. SS, MG, JC-B, and JD: writing—review and editing. All authors read and agreed to the published version of the manuscript.
